# Toddlers’ sensitivity to dominance traits from faces

**DOI:** 10.1038/s41598-023-49385-7

**Published:** 2023-12-15

**Authors:** Cristina-Ioana Galusca, Martial Mermillod, Jean-Claude Dreher, Jean-Baptiste van der Henst, Olivier Pascalis

**Affiliations:** 1 CNRS-Laboratoire de Psychologie et NeuroCognition, Université Grenoble Alpes, BSHM-1251 Av Centrale|CS40700, 38058 Grenoble Cedex 9, France; 2 Centre National de la Recherche Scientifique, Grenoble, France; 3 CNRS-Institut de Sciences Cognitives Marc Jeannerod, UMR5229, Neuroeconomics, Reward, and Decision Making Laboratory; Université Claude Bernard 1, Lyon, Lyon, France; 4 Université Claude Bernard Lyon 1, CNRS, INSERM, CRNL U1028 UMR5292 Trajectoires, Lyon, France

**Keywords:** Human behaviour, Object vision

## Abstract

In adults, seeing individual faces is sufficient to trigger dominance evaluations, even when conflict is absent. From early on, infants represent dyadic dominance relations and they can infer conflict outcomes based on a variety of cues. To date, it is unclear if toddlers also make automatic dominance trait evaluations of individual faces. Here we asked if toddlers are sensitive to dominance traits from faces, and whether their sensitivity depends on their face experience. We employed a visual preference paradigm to study 18- and 24-month-old toddlers’ sensitivity to dominance traits from three types of faces: artificial, male, female. When presented with artificial faces (Experiment 1), 18- and 24-month-olds attended longer to the non-dominant faces, but only when they were in upright orientation. For real male faces (Experiment 2), toddlers showed equivalent looking durations to the dominant and non-dominant upright faces. However, when looking at female faces (Experiment 3), toddlers displayed a visual preference for the upright non-dominant faces at 24 months. To our knowledge, this is the first study to show that toddlers already display sensitivity to facial cues of dominance from 18 months of age, at least for artificial face stimuli.

## Introduction

Human faces are a rich and highly reliable source of information about social categories such as gender, race or age^1,2^. People also use facial traits to evaluate an individual’s *character*, such as how dominant they are. Judging someone’s dominance, understood as the capacity to prevail in conflicts and control others, enables us to decide strategically who to affiliate and share resources with, or alternatively who to avoid due to the physical threat they pose^3^. Brief exposures (~ 100 ms) to faces are sufficient to trigger dominance evaluations who globally show a high consensus across individuals^3–5^. However, research suggests that first impressions are primarily social biases, and may not reflect true character^6,7^. Globally, more masculine features, such as a pronounced jawline and eyebrows^8,9^, and a large facial width-to-height ratio (fWHR) are viewed as more socially dominant and physically stronger, but also as more aggressive and less reliable^10–14^. At a mechanistic level, it has been proposed that these face evaluations are the byproduct of an overgeneralization of the emotion processing from faces^6^. As such, dominant neutral faces bear a subtle structural resemblance to angry faces, which may engender this trait misattribution and similar physiological responses^6,8^.

Research shows that perceived dominance has a crucial role in modulating human social interactions and decision-making. Adults are more likely to follow the gaze of dominant individuals compared to their non-dominant counterparts^15,16^. However, perceived dominance does not impact only low-level automatic behaviours, but high-level decision making processes as well, generally expected to be the result of lengthy rational deliberations. Dominant-looking individuals are more likely to be politically elected^17^, or to be convicted in court^18^.

Dominance impacts social interactions and decisions even in toddlers^19^, thus it is crucial to understand the developmental origins of face evaluations in such a context. When presented with two same-gender faces and asked to judge which one is stronger, preschoolers already choose the individual with more masculine facial features as “stronger”, or more dominant^20–22^. Cogsdill et al.^20^ reported sensitivity to dominance facial traits in 3- and 4-year-old children, and adult-like judgements by 7 years of age. They also showed that young children’s evaluations may be based on more general valence judgements: dominant individuals were judged as mean, and non-dominant individuals as nice. Terrizzi et al.^21^ corroborated these findings by showing that by age 4 children associate physical strength and authority with a dominant face. By the same age, children also see dominant faces as more likely “to go together” with expansive postures or strong bodies, while non-dominant faces as more likely to “go together” with constrictive postures and less powerful bodies^21^. Charlesworth et al.^22^ showed that although children can explicitly judge dominance traits from faces by 3 years of age (e.g., “who is stronger?”), it is not before age 5 that they can make explicit behavior judgements (e.g., “who can pick up really heavy things?”) based on facial traits and adapt their own behavior based on character evaluations (i.e., 5-year-olds prefer to give gifts to non-dominant looking individuals).

What still remains unclear is how children acquire these representations of facial dominance, how early, and whether they are experience-dependent. Previous research suggests that sensitivity to dominance traits from faces may develop more slowly than other dimensions for face evaluation, such as valence or trustworthiness, and may require more extensive face experience^21,23^. One possibility is that sensitivity to dominance traits may develop first for the most frequent face categories (i.e., own race female faces, since women are usually the primary caregivers), and subsequently for less common typologies (i.e., own race male faces). Experience with faces, especially in the first year of life, tunes the face processing system to the most representative exemplars in the environment, such as primary caregiver’s gender^24,25^. A second possibility is that first impressions of facial dominance result from extracting the statistics of the environment and how dominance tends to be associated with masculine traits. If toddlers indeed associate dominance with males, then they may be more sensitive to dominance traits from male-like faces.

The current research aims to fill this gap by exploring the role of social experience on 18- and 24-month-old toddlers’ sensitivity to facial dominance. While the studies cited above only used artificial avatars to explore this capacity, here we used real female and male faces, but also artificial male-like avatars (at the time of testing, female-like avatars were not freely available online). To date, only a handful of studies have investigated the perception of facial dominance in infants^23,26^. Using a looking-time paradigm, this work reported that at 7–8 months of age infants did not spend more time looking at dominant or non-dominant faces while they were sensitive to differences in facial trustworthiness. However, other studies documented that infants are sensitive to dominance when it is introduced by conflicting interactions between two agents. For example, Thomsen et al. ^27^ found that before their first birthday, infants spend more time looking at a situation where a smaller agent outweighs a larger agent, than at a situation in which the larger agent outweighs the smaller one. The authors interpreted this finding as indicating that infants *expect* body size to be a cue to dominance, and that in situations that contradict their expectations, longer looking time reflects greater surprise^27^. Following the same logic, other authors have argued that infants expect an agent with more allies, or placed higher in vertical space, to win in zero-sum conflicts against an agent with fewer allies, or placed lower in space^28,29^.

Moreover, after one year of age infants are not only sensitive to dominance cues but also to the stability of dominance relationships. In particular, when 15-month-old infants are shown a conflict between two geometric social agents, where agent A wins against agent B, they expect the same agent to be dominant in a novel scenario, i.e., they will look longer at the novel scenario if the former subordinate dominant agent prevails over the former dominant agent^30,31^. However, in a similar task using conflicts between two human agents, more complex both at a structural (i.e., more joints, body parts and facial components) and social category level (i.e., gender, age or race) than geometric figures, 18- but not 15-month-olds expected dominance to be a stable relationship across time and different scenarios^32^. This suggests that although early mechanisms of dominance understanding may be in place from the first year of life, extracting and tracking asymmetrical relations from real-world scenarios may be a more challenging task, that children only succeed at towards the end of their second year of life (i.e., after 18 months).

Similar to adults, toddlers also use individual dominance characteristics to guide their social preferences and to make decisions about who to approach or avoid. For example, Thomas et al.^33^ presented toddlers with right-of-way zero-sum conflicts between two puppets, in which one yielded the way for the other to pass. When asked which puppet they preferred, 21- to 31-month-old toddlers consistently chose the dominant puppet, but only when no physical cost was inflicted to the subordinate^33^. Younger infants of 10–16-months, however, consistently chose the puppet who yielded^34^. This suggests that humans initially avoid dominant individuals that may appear threatening and then shift towards a preference for the dominant individual during the second year of life, potentially because they realize that high-status individuals can provide potential benefits^33,35^. So far, though infants and toddlers display great sensitivity to a variety of dominances cues, it is not clear if this also includes dominance-related information from facial features. The evaluation of facial dominance might differ from the evaluation of relational dominance, and the mechanisms involved in judging and tracking them may be distinct too. While a dominant face may imply threat and meanness, a dominant behavior may be a sign of social success.

Our primary goal was to determine whether toddlers are sensitive to dominance traits from faces. The current study investigated toddlers’ (18- and 24-month-olds) spontaneous visual preference presented two faces same-gender faces that differed in dominance. Previous studies using a preferential looking paradigm reported that 7-month-olds displayed a preference for trustworthy faces over untrustworthy faces, but showed no preference for faces varying in dominance^23^; see also Ref. ^26^ for similar findings. Authors argued that trust evaluations may precede dominance evaluations because of their importance in judging who is friend and who is foe^36^. Though previous research documents an early sensitivity to trustworthiness facial cues in the first year, it is unclear when infants become sensitive to facial dominance. In three experiments, we used an implicit preferential looking task with no training and presented different types of faces (artificial faces in Experiment 1, male faces in Experiment 2, and female faces in Experiment 3), to test three opposing hypotheses. First, if facial dominance evaluations are primarily driven by early experience with faces overall, then toddlers should be more sensitive to dominance traits for female faces (i.e., highly familiar), compared to male (i.e., moderately familiar) or artificial faces (i.e., male-like highly unfamiliar).

A second hypothesis is that toddlers may display a higher sensitivity to dominance traits from male-like faces. Males tend to display more verbal and non-verbal dominant behaviour. For example, men adopt more dominant postures than women^37^. They also have a more dominant gaze behavior, and display more direct gaze when speaking and averted gaze when listening^38^. Finally, stereotypical female faces are perceived as less dominant than stereotypical male faces by adults^39^, which may be reinforced by gender stereotypes leading to considering women as less strong and dominant compared to men^36^.

A third possibility is that early sensitivity to facial dominance does not depend on toddlers’ different experience with male and female faces, but rather on adaptive face-processing mechanisms, which enable them to recognise emotions and infer character traits from these emotions. For instance, adults consider angry and happy faces to have high dominance, while they consider fearful and sad faces to have low dominance^6,40^. According to the overgeneralisation hypothesis, first impressions are generated by an adaptive mechanism that over-reads emotional content even from neutral faces, which explains why trait judgements are often inaccurate^9^. By their first birthday, infants can recognise basic facial emotions, categorise them and adapt their behaviour based on their caregivers’ emotional expressions^41^. Though infants display an advantage in processing female faces in the first months^41^, no studies to date documented better emotion perception for female compared to male faces in toddlers. Based on these studies together, a third prediction is that toddlers would display similar sensitivity to facial dominance for male and female faces.

Finally, if toddlers displayed a selective visual preference between dominant and non-dominant faces, we expected them to prefer non-dominant faces, because dominant characters are generally perceived as more threatening and aggressive^4^. Previous research showed that when presented with happy and angry faces, infants attend longer to the happy faces. This has been interpreted as a visual avoidance mechanism of negative affect and a general preference for positively valenced social information^42^. In line with these findings, Thomas et al. also found that in explicit social preference tasks toddlers preferred the non-dominant social agent when the dominant inflicted force^33^, confirming this overall tendency to avoid social contact, even if only visually, with threatening individuals.

## General method

We used a paired visual preference paradigm, where infants viewed pairs of faces selected to be one high and one low in perceived dominance, but equivalent in attractiveness and masculinity. Prior to the toddler study, we conducted a norming study on French adults, based on which face stimuli were selected for the toddler experiments. Real faces were taken from the Karolinska Directed Emotional Faces (KDEF) database^43^. We selected 16 female and 16 male faces displaying a neutral facial expression and direct gaze towards the camera. Artificial faces were taken from a database of synthetic faces whose perceived dominance was varied parametrically. These faces were generated using FaceGen, using data-driven computational models^4,44,45^. The artificial faces (N = 14 pairs) were all male-like, and each identity pair had a low dominance (0 SD) and a high dominance face (+ 3 SD).

The pre-selected real and artificial faces were rated by 20 French adult volunteers on three traits of interest: dominance, attractiveness and masculinity/femininity using a 1–9 Likert scale, where 1 was “*Not at all [insert trait]*” and 9 was “*Extremely [insert trait]*”. Pairs of faces were selected such that one was high on dominance one was low on dominance (z-scores more than 2 SDs apart for each pair), but each pair displayed faces with equivalent levels of attractiveness and masculinity (z-scores less than 1 SD apart for each pair; see Table 1 for details). As such, each pair contained a dominant and non-dominant exemplar, and here what we consider to be a dominant face is simply a face with high ratings on a dominance scale by adults, while a non-dominant face is a face rated low on dominance by adults.

**Table 1:** Standardized z-scores for the face items used in each experiment, rated on three facial dimensions: dominance, attractiveness and masculinity.

Experiment	Face item	Dominance	Attractiveness	Masculinity
1 Artificial faces	High dominance	1.986	− 0.608	1.111
	Low dominance	− 0.307	− 0.260	0.237
	High dominance	1.678	− 1.738	1.111
	Low dominance	− 0.553	− 1.173	0.139
2 Male faces	High dominance	1.686	1.018	0.854
	Low dominance	− 1.694	0.869	0.197
3 Female faces	High dominance	1.275	− 1.292	0.332
	Low dominance	− 1.31	− 0.568	0.087

**Figure 1:**
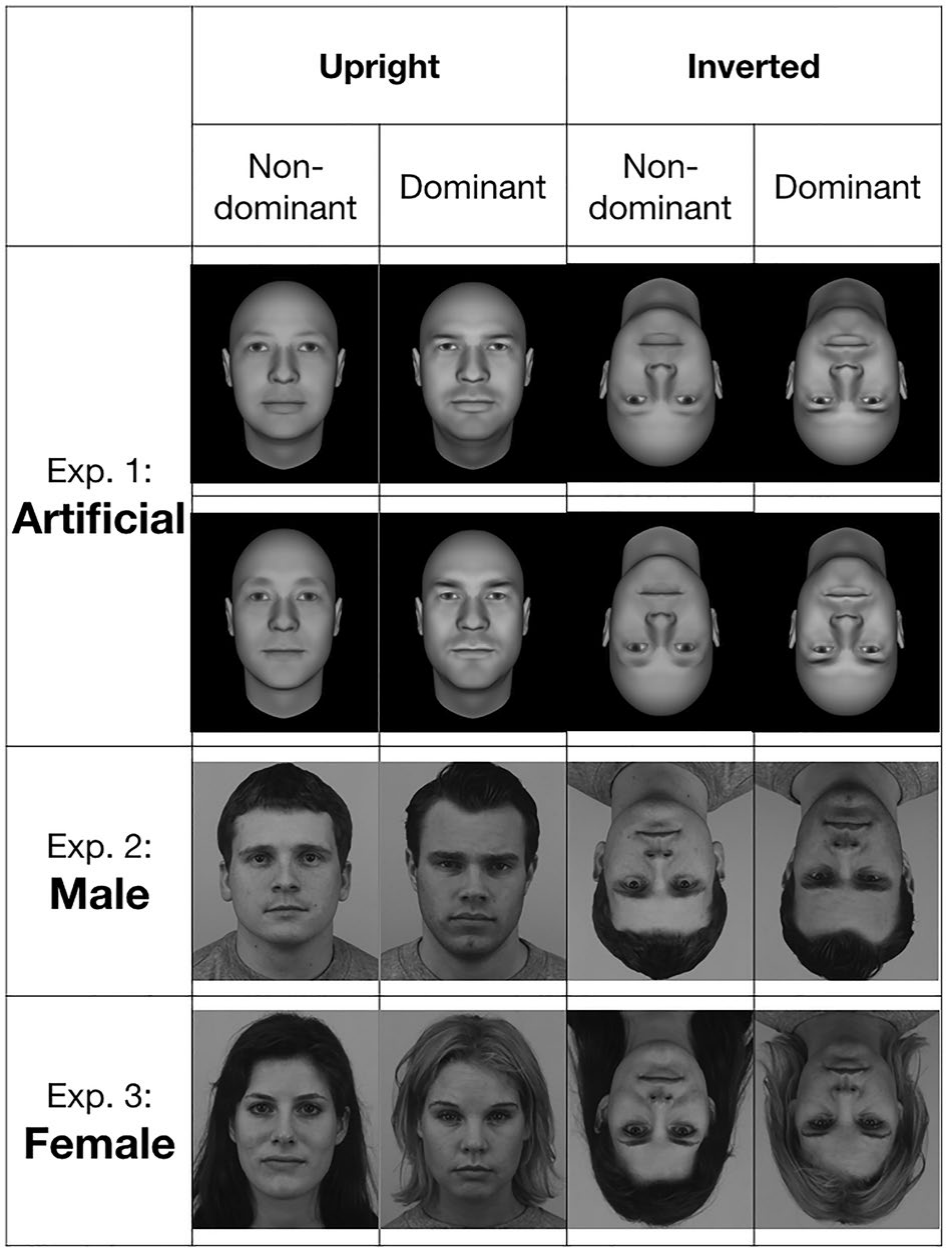
Pairs of face stimuli used in Experiments 1–3 for the upright and inverted orientations.

The selected faces (2 pairs of artificial faces and 2 pairs of real faces, see Fig. 1) were resized to create same height face pairs, and were matched in luminosity using a custom-made script in Matlab. First, images were converted from RGB to grayscale. Second, the average luminosity for every face was calculated in digital value. We set the average luminosity to 110, which was the average luminosity of all the images, and then computed a correction factor for each of them. Finally, the luminosity of each image was corrected to this average value, which yielded pairs of face images matched in luminosity.

## Experiment 1

This experiment evaluated toddlers’ sensitivity to dominance traits from male-like artificial faces using a paired visual preference paradigm.

### Methods

#### Participants

A total of 65 participants were tested: 33 full-term 18-month-olds (19 females; M age = 563 days; SD = 19.8 days; age range 526–623 days), 31 full-term 24-month-olds (14 females; M age = 771 days; SD = 38.6 days; age range 717–863 days). Data collection occurred in France where data on race/ethnicity cannot legally be collected. Since our study investigated the role of exposure to female and male faces on trait judgement, we used parental report to evaluate infants’ experience with faces. For the 18-month-olds, the mean percentage of time exposed to adult female faces was 72%, where 32 out 33 participants spent more than 50% of their awake time with women. The 24-month-olds were exposed to female faces 71% of the time, with 30 out of 31 participants exposed more than half their time to females. This is in agreement with our knowledge about toddlers’ early experience and the fact that they are mainly exposed to female faces. The present study was conducted in accordance with guidelines laid down in the Declaration of Helsinki. All parents gave their informed consent prior to their infants' participation in the study. This paradigm was approved by the Ethical Committee at the Université Grenoble Alpes in France (CERGA IRB00010290‐2018‐02‐06‐39).

#### Stimuli and procedure

Infants were tested in a quiet chamber at the Babylab Grenoble, Université Grenoble Alpes. Infants sat on their parents’ lap approximately 60 cm away from a screen, which displayed the images. The experimenter was out of sight during testing, and parents and the experimenter remained quiet. We used a paired visual preference task to test infants’ spontaneous visual preference for dominant or non-dominant artificial faces. Each pair of photographs was presented for a total duration of 10 s. Based on adult ratings (see “General method” above), we selected two pairs of artificial faces to use in this experiment. Each infant was presented with four trials (four pairs of images), half presenting the face pairs upright and half presenting the inverted faces. The inverted trials were introduced as a control for other low-level visual features that may drive visual attention (e.g., luminance, shape). Each participant saw two trials per condition, to counterbalance the left–right positioning of the photographs. The order of presentation of each type of trials (upright or inverted) was counterbalanced across infants. Half of the infants started with the upright condition, and the other half started with the inverted stimuli. We recorded infant looking behavior to the stimuli, and the videos were analyzed offline frame by frame on a computer using specialized software. An independent observer recoded 20% of the data for reliability. Both observers were blind to condition. The average level of inter-observer agreement across the two age groups was very high (Pearson *r* = 0.95).

### Results

First, infants’ looking behavior was analyzed separately for each age group (18-month-olds and 24-month-olds) and face orientation (upright or inverted). The analyses for each trial were conducted on Difference Scores, which quantify infants’ visual preference for a dominant or non-dominant face by calculating the difference in total looking time to each of the two faces divided by the total looking time to the two faces: (LT_Dominant_—LT_Non-dominant_)/(LT_Dominant_ + LT_Non-dominant_). In our analyses, scores above 0 indicate an overall preference for dominant faces, while scores below 0 indicate a preference for the non-dominant faces. Trials where participants showed a side bias (i.e., they only looked towards one of the faces) were excluded from the analyses (N = 1). In a second analysis we tested the omnibus effects of age and face orientation on infants’ looking preferences. For follow-up Bayesian analyses, see the Supplementary Material S1.

#### 18-month-olds

**Figure 2:**
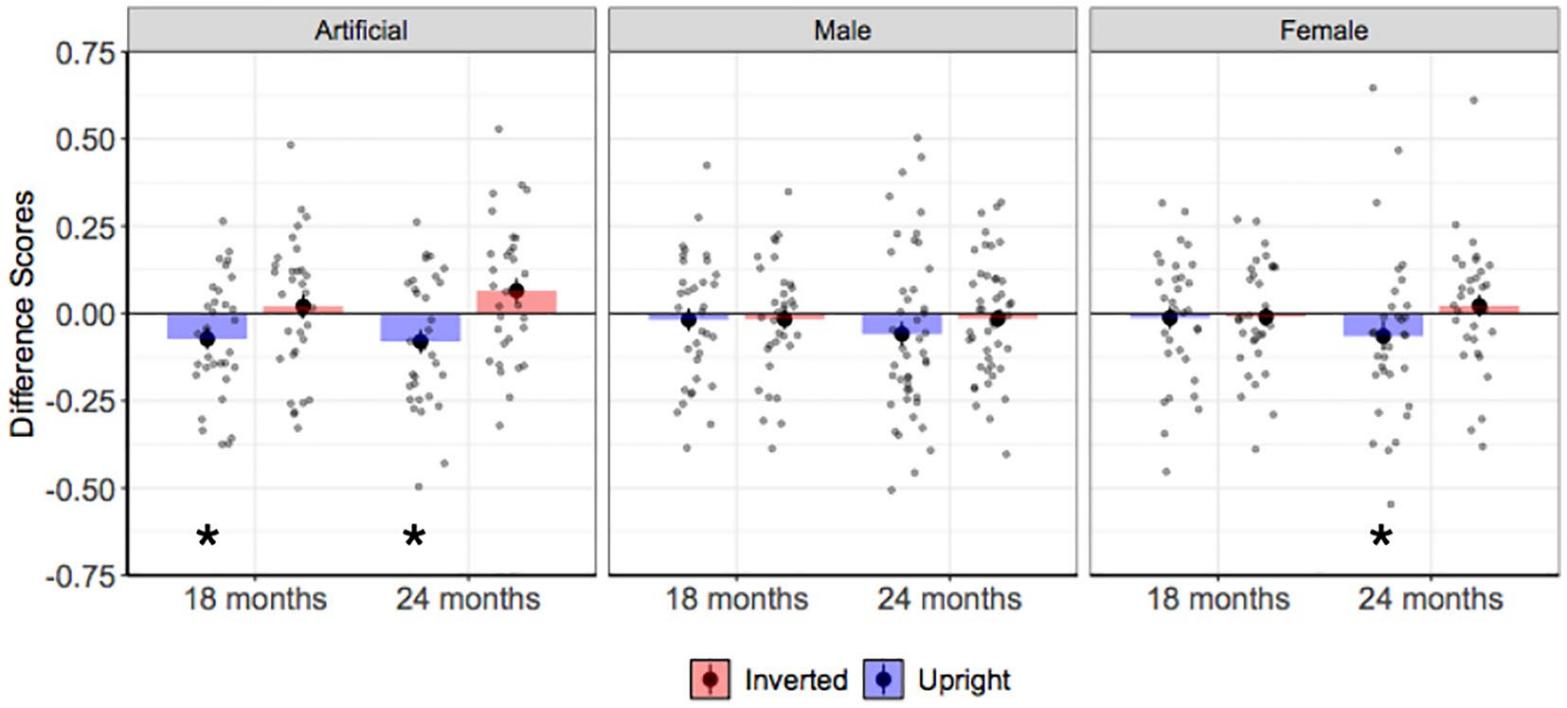
Difference scores for Experiments 1–3. Negative difference scores indicate a preference for non-dominant faces. Positive difference scores indicate a preference for dominant faces. Stars indicate a significant difference at 0.05 level between difference scores and the 0% chance level. Error bars represent the standard error of the mean.

We used a Wilcoxon signed-rank test to analyze toddlers’ preference for the *upright* dominant or non-dominant artificial faces by comparing their difference scores against the 0% chance level. This analysis revealed that 18-month-old infants looked significantly longer at the non-dominant compared to the dominant artificial faces when they were upright (*p* = 0.033; *Difference Score* = − 0.073; *SE* = 0.029; see Fig. 2). However, the analysis on infants’ looking behavior towards the inverted faces revealed that they spent a comparable amount of time looking at the inverted non-dominant and dominant faces (*p* = 0.48; *Difference Score* = 0.021; *SE* = 0.033; see Fig. 2). Finally, a Wilcoxon signed-rank test comparing 18-month-olds’ Difference Scores to the upright and inverted faces revealed that toddlers looked significantly longer at the non-dominant artificial face in its upright compared to its inverted orientation (*p* = 0.029).

#### 24-month-olds

A Wilcoxon signed-rank test comparing difference scores against the 0% chance level revealed that 24-month-old infants looked significantly longer at the non-dominant compared to the dominant artificial faces (*p* = 0.023; *Difference Score* = − 0.080; *SE* = 0.033; see Fig. 2). The analogous analysis on infants’ looking behavior to inverted artificial faces revealed that they spent a comparable amount of time looking at the inverted non-dominant and dominant faces (*p* = 0.094; *Difference Score* = 0.065; *SE* = 0.035; see Fig. 2). A Wilcoxon signed-rank test comparing Difference Scores to the upright and inverted faces revealed that 24-month-olds looked significantly longer at the non-dominant artificial face in its upright compared to its inverted orientation (*p* = 0.015).

#### All ages and face orientations

We then explored the overall effect of age and face orientation on infants’ preferential attention to dominant over non-dominant faces. We used a linear mixed-effects model to analyse Difference Scores with R (R Core Team, 2012). Participants were random factors, while Age (2: 18 and 24 months) was a categorical between-subjects fixed factor, and Face Orientation (2: Upright and Inverted) was a categorical within-subjects fixed factor. In line with our previous results, statistical analyses yielded a significant effect of Face Orientation (Estimate = − 0.060, SE = 0.0.016, *t* = − 3.665, *p* < 0.0004), showing that toddlers preferred to look at non-dominant faces but only when faces were displayed upright. No other main effect or interaction were significant (t < 1).

### Discussion

In summary, we found that by 18 months of age toddlers displayed a visual preference for non-dominant artificial faces compared to their dominant counterparts. Presenting the same pairs of faces in inverted orientation abolished the effect, which suggests that the differences found in the upright orientation were due to processing the dominance facial traits, and likely discriminating between dominant and non-dominant features, rather than other low-level differences between images which were maintained when inverted.

Our results add to previous studies that showed that at 3 years of age children can *explicitly* judge dominance (i.e., “who is stronger?”) based on facial traits of artificial avatars^20^. Likewise, the current experiment used artificial male-like faces where only dominance-related facial features were manipulated (e.g., jaw line, eyebrows, fWHR), while face identity and other characteristics were kept constant (e.g., hairline, eye color, nose shape). Nevertheless, faces in the real-world are a lot more diverse. Do toddlers also show visual preferences on the basis of dominance traits when shown two different real faces? Considering the asymmetry in experience with female (highly familiar and numerous exemplars) and male faces (less familiar and less exemplars), we tested toddlers’s preferential looking to dominant and non-dominant faces for each gender in different experiments. In Experiment 2, we tested toddlers’ sensitivity to dominance facial traits from real male faces.

## Experiment 2

Here we evaluated toddlers’ sensitivity to dominance traits from real male faces. Experiment 1 showed that 18 and 24-month-old toddlers look longer at non-dominant compared to dominant faces, when artificial, male-like stimuli were used, and dominance unrelated features were highly controlled within each pair. The current experiment aims to extend these findings to more realistic scenarios, where faces are more variable, also along dimensions unrelated to dominance. This study presented infants with pairs of different real male faces matched in attractiveness and masculinity, but mismatched in dominance.

### Methods

#### Participants

A total of 80 participants were tested: 33 full-term 18-month-olds (13 females; M age = 563 days; SD = 29.1 days; age range 526–629 days), 46 full-term 24-month-olds (21 females; M age = 766 days; SD = 38 days; age range 717–863 days). Data collection occurred in France where data on race/ethnicity cannot legally be collected. The present study was conducted in accordance with guidelines laid down in the Declaration of Helsinki. All parents gave their informed consent prior to their infants' participation in the study. This paradigm was approved by the Ethical Committee at the Université Grenoble Alpes in France (CERGA IRB00010290‐2018‐02‐06‐39).

### Results and discussion

As in Experiment 1, we first analysed toddlers’ looking behavior (i.e., Difference Scores) for each age group and face orientation separately. Subsequently, we conducted an omnibus analysis of their attention to dominant and non-dominant male faces. Trials where participants showed a side bias (no looks towards one of the faces) were excluded from the analyses (N = 3). For additional follow-up Bayesian analyses, see the Supplementary Material S1.

#### 18-month-olds

We used a Wilcoxon signed-rank test to analyze toddlers’ preference for the *upright* dominant or non-dominant real male faces by comparing their difference scores against the 0% chance level. This analysis revealed that 18-month-old infants looked for an equivalent duration at the non-dominant and dominant real male faces (*p* = 0.589; *Difference Score* = − 0.019; *SE* = 0.032; see Fig. 2). Similarly, the analysis on infants’ looking behavior towards the inverted faces no visual preference for the inverted non-dominant and dominant male faces (*p* = 0.673; *Difference Score* = − 0.017; *SE* = 0.029; see Fig. 2).

#### 24-month-olds

A Wilcoxon signed-rank test comparing difference scores against the 0% chance level revealed that 24-month-old infants looked for an equivalent duration at the non-dominant compared to the dominant male faces (*p* = 0.273; *Difference Score* = − 0.031; *SE* = 0.036; see Fig. 2). The analogous analysis on infants’ looking behavior to inverted male faces revealed that they spent comparable durations of time looking at the inverted non-dominant and dominant male faces (*p* = 0.897; *Difference Score* = 0.002; *SE* = 0.023; see Fig. 2).

#### All ages and face orientations

We used a linear mixed-effects model to analyse Difference Scores and we declared participants as random factors, Age (2: 18 and 24 months) as a categorical between-subjects fixed factor, and Face Orientation (2: Upright and Inverted) as a categorical within-subjects fixed factor. These statistical analyses yielded no significant main effect or interaction (t < 1).

We found no evidence that toddlers preferred real male faces based on their dominance traits, when images were presented in a canonical or inverted orientation. When using images of real faces, we could not replicate our findings from Experiment 1. This suggests that although toddlers *can* display visual preferences based on very brief exposures to faces, this mechanism is not yet mature and hinges on the task being simplified by emphasising dominance diagnostic features and minimising dominance non-diagnostic features, as was the case for the artificial male-like avatars. However, another approach for simplifying toddlers’ task is to present them with highly familiar real faces they have expertise in processing, such as female faces. Due to their increased exposure to females, from their first year of life infants have superior processing abilities for female compared to male faces^25,46^. For example, while infants can discriminate unfamiliar female faces by 3 months of age^47^, they still have difficulty discriminating between unfamiliar male faces at 7 months of age^48^. To test whether face familiarity facilitates toddlers’ facial dominance evaluation, in Experiment 3 we presented them with pairs of real female faces, matched in attractiveness and masculinity.

## Experiment 3

When presented with pairs of male faces mismatched in levels of dominance, toddlers looked longer at non-dominant individuals when they saw artificial male-like avatars (Experiment 1) where dominance traits were exacerbated, but showed no visual sensitivity to facial dominance from real male faces (Experiment 2). If experience with a face category facilitates toddlers’ face trait judgements, we predicted better sensitivity to character traits for real female compared to real male faces, because female faces are more frequent in their environment in the first 2 years of life^49^. Thus, Experiment 3 evaluated toddlers’ implicit sensitivity to dominance traits from female faces, otherwise matched in attractiveness and masculinity.

### Methods

#### Participants

We tested a total of 68 participants: 33 full-term 18-month-olds (20 females; M age = 560 days; SD = 22.5 days; age range 526–623 days), 33 full-term 24-month-olds (11 females; M age = 770 days; SD = 37.3 days; age range 717–863 days). Data collection occurred in France where data on race/ethnicity cannot legally be collected. For the 18-month-olds, the mean percentage of time exposed to adult female faces was 68%, where 28 out 33 participants spent more than 50% of their awake time with women. The 24-month-olds were exposed to female faces 71% of the time, with 31 out of 33 participants were exposed more than half their time to females. The present study was conducted in accordance with guidelines laid down in the Declaration of Helsinki. All parents gave their informed consent prior to their infants' participation in the study. This paradigm was approved by the Ethical Committee at the Université Grenoble Alpes in France (CERGA IRB00010290‐2018‐02‐06‐39).

### Results and discussion

As in Experiment 1, we first analysed toddlers’ looking behaviour (i.e., Difference Scores) for each age group and face orientation separately. Subsequently, we conducted an omnibus analysis of their attention to dominant and non-dominant female faces. For follow-up Bayesian analyses, see the Supplementary Material S1.

#### 18-month-olds

We used a Wilcoxon signed-rank test to analyze toddlers’ preference for the *upright* dominant or non-dominant female faces by comparing their difference scores against the 0% chance level. This analysis revealed that 18-month-old infants looked for an equivalent duration at the non-dominant and dominant female faces (*p* = 0.944; *Difference Score* = − 0.013; *SE* = 0.032; see Fig. 2). Similarly, the analysis on infants’ looking behavior towards the inverted faces show no visual preference for the inverted non-dominant and dominant female faces (*p* = 0.819; *Difference Score* = − 0.001; *SE* = 0.027; see Fig. 2).

#### 24-month-olds

A Wilcoxon signed-rank test comparing difference scores against the 0% chance level revealed that 24-month-old infants looked significantly longer at the non-dominant compared to the dominant female faces (*p* = 0.033; *Difference Score* = − 0.065; *SE* = 0.041; see Fig. 2). The analogous analysis on infants’ looking behavior to inverted female faces revealed that they spent comparable durations of time looking at the inverted non-dominant and dominant female faces (*p* = 0.313; *Difference Score* = 0.022; *SE* = 0.032; see Fig. 2). A Wilcoxon signed-rank test comparing Difference Scores to the upright and inverted faces revealed that 24-month-olds looked marginally longer at the non-dominant female face in its upright compared to its inverted orientation (*p* = 0.090).

#### All ages and face orientations

We explored the overall effect of age and face orientation on infants’ preferential attention to dominant over non-dominant female faces. We conducted a linear mixed-effects model where participants were a random factor, Age (2: 18 and 24 months) was a categorical between-subjects fixed factor, and Face Orientation (2: Upright and Inverted) was a categorical within-subjects fixed factor. No main effects or interactions were significant (t < 1.5).

To sum up, we found that by 24 months of age toddlers attended longer to the non-dominant compared to the dominant real female faces. Presenting the same faces in inverted orientation abolished this effect, which indicated that the differential effects were due to how toddlers processed the facial traits in their canonical orientation, and not other low-level effects that may have driven their attention. Toddlers’ extensive exposure to female faces in their first years of life may be responsible for their ability to detect even subtle cues of facial dominance from female faces.

### Exploratory analyses

#### Comparison between male and female faces (Experiments 2 and 3)

To directly test our hypothesis that face experience boosts sensitivity to dominant traits from familiar types of faces, we conducted an exploratory analysis on 24-month-old toddlers’ Difference Scores, taking into account the gender of images. We tested a linear mixed-effects model with participants as a random factor, FaceType (2: Male and Female) as a categorical between-subjects fixed factor, Face Orientation (2: Upright and Inverted) as a categorical within-subjects fixed factor, and Difference Score as the dependent variable. This analysis revealed a marginal effect of Face Orientation (p = 0.082), as 24-month-olds looked longer to the upright non-dominant faces, compared to the inverted real faces. No significant difference between male and female faces was found and no interaction (t < 1).

Overall, 24-month-old toddlers displayed a visual preference for non-dominant faces, with looking behavior for male and female faces trending in the same direction. Additionally, toddlers attended marginally longer to the non-dominant faces only when they were displayed in upright orientation, suggesting their visual response was likely driven by facial traits, rather than low-level image features.

#### Correlation face experience and visual preference

**Figure 3:**
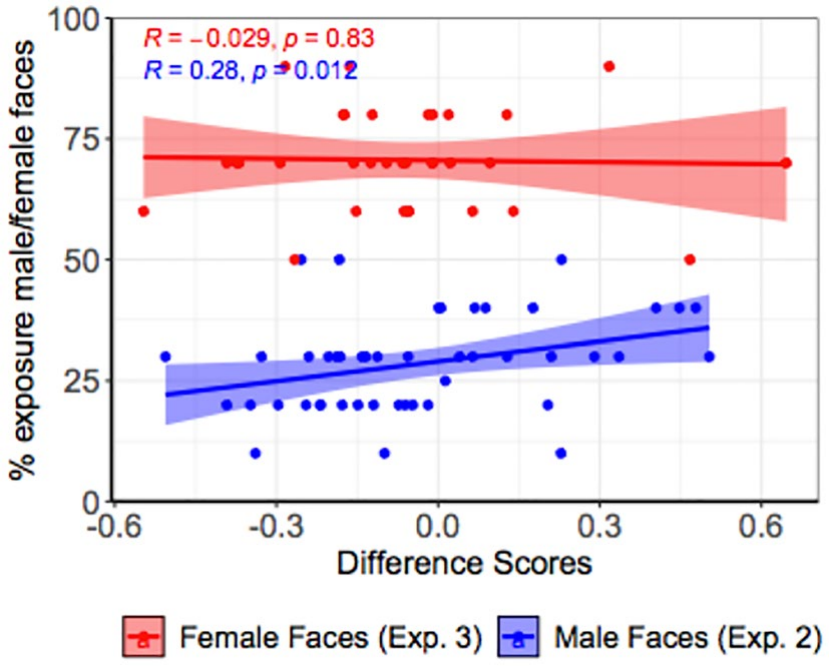
In Experiment 2, toddlers with a higher exposure to male faces showed a stronger visual preference for dominant individuals, as revealed by a significant Kendall correlation (τb = 0.28, p = 0.012). However, in Experiment 3, we found no relation between toddlers’ exposure to female faces and their visual preferences. Mean difference scores are plotted for all participants with jitter.

To further explore the relationship between toddler experience with female and male faces and their visual preferences for faces differing in dominance traits, we conducted Kendall’s correlations for the male and female faces separately. For each type of face, we correlated difference scores to the percentage of exposure to that particular type of face. In Experiment 2, we found a significant Kendall correlation between Face experience and Difference scores (τb = 0.28, p = 0.012; see Fig. 3). This suggests that toddlers with a higher exposure to male faces showed a stronger visual preference for dominant individuals. However, in Experiment 3, we found no significant correlation between Face experience and looking time towards female faces (τb = − 0.029, p = 0.83; see Fig. 3).

One important aspect to keep in mind when interpreting the direct relation between experience with faces and visual preferences is that participants in our sample were exposed to female faces at least 50% of the time. Therefore, a “high” exposure to male faces in this case means 40–50% of the time.

Masculine facial traits are generally correlated with impressions of dominance in adults^9,50^, and infants too may represent a prototypical female face as non-dominant, and a prototypical male face as dominant. Thus, when presented with male faces, infants with a more balanced exposure to female and male faces may prefer to attend to the more prototypical male face (i.e. dominant preference), while when those with a limited exposure to male faces may be driven by the female face prototype, for which they have a stronger representation (i.e., non-dominant preference). However, it is important to note that an important limitation of this analysis is that parental reports of face familiarity may not be a very sensitive measure (e.g., a majority of parents declared that their infants were exposed to women 70% of the time). More detailed questionnaires and scores taking into account the number of women/men infants are regularly in contact with may be more suitable to capture the impact of perceptual experience on first impressions from faces. For this reason, our interpretation of this correlation remains limited for now and further studies are needed to understand the role of face familiarity in infants’ evaluations of facial dominance.

## General discussion

Across three experiments, we examined the developmental pathway of toddlers’ sensitivity to dominance traits from faces by implicitly measuring their spontaneous preference for dominant and non-dominant artificial male-like faces (Experiment 1), real male faces (Experiment 2) and real female faces (Experiment 3). Our results revealed that, at 18 months of age, infants preferred non-dominant faces, but only for upright artificial male-like faces, where dominance features were magnified. Inversion cancelled this effect. For more complex real stimuli, where faces differed on a variety of other features other than dominance, toddlers showed sensitivity to dominance facial traits at 24 months, but not at 18 months. They displayed this sensitivity only for female faces, when we contrasted upright with inverted faces. At this age toddlers are more exposed to female faces and their superior experience with female faces is two-fold: (1) infants are exposed to more female face exemplars than male face exemplars (e.g., several female instructors in daycare), and (2) they see female faces for a higher proportion of time than male faces (e.g., toddlers in our sample spent an average of 70% of time with women, and only 30% of time with men). Prior research showed that explicit dominance face-trait evaluations are consistently above-chance from 3 years of age^20,22^, while other studies found no sensitivity to dominance facial traits in early infancy^23,26^. To our knowledge, this is the first study to show sensitivity to facial dominance before age 2. This early sensitivity to facial dominance supports previous proposals that infants pay special attention to cues of dominance, such as physical size, group size or vertical position, and use these cues to make inferences about others’ behavior and their capacity to prevail in conflicts^27–29^.

### Dominance-based preferences for artificial faces precede those for real faces

Here, toddlers looked longer at the non-dominant face at 18 months for artificial faces and became sensitive to dominance traits from real female faces at 24 months. Additionally, our exploratory analyses showed a marginal visual preference for non-dominant real faces of both genders when they were analysed together. However, from the three types of faces used in this study, artificial faces were the least familiar to toddlers. So what may account for the earlier sensitivity to artificial avatars compared to real faces? One possibility may be that each pair of faces had the same identity and differed only in their perceived facial dominance, conveyed by differences in their jawline, eyebrows and the overall fWHR. An additional advantage of these avatars is that they have been extensively validated and used in adult and child research^4,45^. One potential avenue for future research is to test whether toddlers maintain their sensitivity to dominance facial traits even for pairs of avatars of different identities (i.e., that display more facial trait differences along dimensions irrelevant for dominance evaluations).

A second potential explanation is that a series of low-level differences between artificial and real faces may have simplified toddlers’ task in the first experiment. For instance, artificial faces displayed no hair and were presented against a black background, unlike female and male faces who had different haircuts and were displayed against a grey background. The contrasting background and the lack of hair for the artificial faces may have removed potential distractors and directed toddlers’ attention to the specific facial features that differed between the avatars in each pair—the more or less prominent jawline, the higher or lower facial width-to-height ratio—which are the exact features used (at least by adults) to judge dominance. Thus, we found an effect for artificial faces that carefully controlled the variability of other perceptual features than those related to dominance, but also for ecological stimuli for which other perceptual features are random (i.e., vary according to how those features appear in daily life situations). Future research should investigate if removing the hair for real faces boosts toddlers’ sensitivity to dominance traits. Finally, a third factor that may explain why toddlers displayed dominance-based visual preferences for artificial faces before real faces is that the avatars account for a larger variability in the population than two real exemplars, since they are generated based on ratings for a large set of real faces.

### Dominance-based visual preferences for real male and female faces

In Experiment 3, toddlers looked longer at non-dominant female faces at 24 months. Although toddlers showed the same trend when presented with male faces in Experiment 2, this result did not reach significance. However, toddlers’ visual preference scores for upright female faces were not significantly different from those for male faces at 24 months. Even though, based on parental estimations, children in our sample were exposed more than double their awake time to female faces compared to male faces (i.e., 70% of the time to female faces, 30% of the time to male faces), we have no evidence that the frequency of exposure drives their early sensitivity to dominance traits. In fact, cross-cultural research on adults’ evaluation of faces also brings into question the perceptual expertise account (i.e., character judgements of faces are driven by exposure to faces), because first impressions from faces are rather coherent across different cultures and populations^51–53^. Despite language differences that shape how traits are described, the underlying psychological attributes used to represent these traits may be shared across cultures. Other studies, however, suggest that there is more cross-cultural correspondence for traits such as approachability, compared to capability-based social traits^54^. Moreover, when adults have to spontaneously label their first impressions from faces, dominance is less often mentioned than attractiveness or trustworthiness^4^, even when the faces displayed are clearly dominant. For these highly dominant exemplars, adults usually employ competence-related words to describe then, such as intelligence, and their ratings are less consistent and more variable across cultures^39^. Thus, attractiveness and trustworthiness may be a primary dimension for social evaluation^36^, while dominance and competence may be less stable, and more dependent on context. This is consistent with previous research showing that infants are sensitive to valence or trustworthiness traits from faces, before they acquire this ability for dominance traits, which require more extensive face experience^23,26^.

### Early visual preference for non-dominant others

Importantly, toddlers in the current study showed a consistent visual preference for non-dominant faces, for the artificial and female exemplars (Experiments 1 and 3). One possible interpretation is that toddlers prefer to attend to non-dominant faces because they are more similar to the prototype of their primary caregiver (i.e., non-dominant faces may appear to them more feminine), and thus, more familiar to them than male-like faces. However, our stimuli were matched in masculinity, based on adult ratings.

If toddlers’ attentional pattern was not driven by familiarity/femininity, another possibility is that toddlers looked longer at the stimuli they found less threatening. The same pattern of visual preference has been previously documented in younger infants presented with emotional faces. For example, 7- and 12-month-old infants preferred to look at happy compared to angry faces^42^, similar to their younger 4- and 6-month-old peers^55^. In our study, the non-dominant faces were neutral, which makes it unlikely that infants attended longer to them for rewarding and positive experiences, as in the case of happy faces. One way to interpret our results is that toddlers may have perceived dominant faces as more aggressive and physically threatening, which triggered their avoidance behavior. Dominant facial traits are strongly correlated to those of threat or aggression^3^, and linked to emotional facial expressions: non-dominant faces appear more fearful, while dominant faces appear angrier. Studies in social referencing further support this interpretation. When infants witness positive, negative or neutral facial expressions towards an object, they touch or explore a lot less those negatively referenced than those positively or neutrally referenced^56^.

The threat avoidance interpretation of our current findings is also consistent with an explicit task carried out with 21–31-month-old toddlers, who used abstract figures to present a dominance relation between two social agents. Toddlers had to choose between the winner and the loser of a zero-sum conflict, and they generally preferred the winner. However, when the winner inflicted force on the loser and posed a physical threat to them, infants switched their preference to the non-dominant individual^33^. This explicit avoidance of threatening agents is coherent with the spontaneous visual preference for non-dominant faces reported here.

### Future directions—neural responses

Recent fMRI studies in human infants and non-human primates show that the large-scale brain organisation of faces in the visual cortex and infero-temporal cortex is adult-like within a few months after birth and is subsequently refined through development^57,58^. Some functional organisation is present at 1 month, and face-selective patches emerge over the first year of development^58^. Regarding the dynamics of the neural processes engaged in representing facial dominance, a recent ERP study reported that dominance levels did not modulate an early component of face processing, known as the N170 component, but did modulate the late positive potential (LPP)^59^. This suggests that dominance, as other hierarchical cues, is evaluated at a later stage of face processing^60,61^. In infants, ERP studies are needed to investigate the dynamics of face representation, perhaps by investigating two components that have been proposed as possible “developmental precursors of the adult N170”, namely the N290 and P400^62,63^.

## Conclusion

Making rapid and automatic character judgements based on facial traits is a pervasive social bias that affects our choices, even in situations when our decisions are believed to be rational (e.g., electoral voting^17^). Prior research suggests that face-to-trait judgements of dominance are consistently above chance at 3 years of age, and reach adult-like levels by 5–6 years of age^20–22^. Here we demonstrate a much earlier sensitivity to dominance traits from faces, starting at 18 months for artificial avatars and at 24 months for real female faces. This suggests that children may not need regular exposure to a large number of face exemplars to develop sensitivity to dominance traits^64^. This early emerging preference for non-dominant faces suggests that trait information may begin to impact children’s social preferences and behavior much earlier than previously thought. Future research should explore toddlers’ expectations of individual behavior or physical traits based on facial appearance.

### Supplementary Information

Supplementary Information.

## References

[1] Calder AJ, Young AW (2005). Understanding the recognition of facial identity and facial expression. Nat. Rev. Neurosci..

[2] Locke V, Macrae CN, Eaton JL (2005). Is person categorization modulated by exemplar typicality?. Soc. Cogn..

[3] Todorov A, Said CP, Engell AD, Oosterhof NN (2008). Understanding evaluation of faces on social dimensions. Trends Cogn. Sci..

[4] Oosterhof NN, Todorov A (2008). The functional basis of face evaluation. Proc. Natl. Acad. Sci..

[5] Todorov A, Olivola CY, Dotsch R, Mende-Siedlecki P (2015). Social attributions from faces: Determinants, consequences, accuracy, and functional significance. Annu. Rev. Psychol..

[6] Said CP, Sebe N, Todorov A (2009). Structural resemblance to emotional expressions predicts evaluation of emotionally neutral faces. Emotion.

[7] Willis J, Todorov A (2006). First impressions: Making up your mind after a 100-ms exposure to a face. Psychol. Sci..

[8] Hess U, Adams RB, Kleck RE (2009). The categorical perception of emotions and traits. Social Cognit..

[9] Zebrowitz LA, Montepare JM (2008). Social psychological face perception: Why appearance matters. Social Personality Psychol. Compass.

[10] Toscano H, Schubert TW, Sell AN (2014). Judgments of dominance from the face track physical strength. Evolut. Psychol..

[11] Carré JM, McCormick CM, Mondloch CJ (2009). Facial structure is a reliable cue of aggressive behavior. Psychol. Sci..

[12] Stirrat M, Perrett DI (2010). Valid facial cues to cooperation and trust: Male facial width and trustworthiness. Psychol. Sci..

[13] Stirrat M, Perrett DI (2012). Face structure predicts cooperation: Men with wider faces are more generous to their in-group when out-group competition is salient. Psychol. Sci..

[14] Geniole SN, Denson TF, Dixson BJ, Carré JM, McCormick CM (2015). Evidence from meta-analyses of the facial width-to-height ratio as an evolved cue of threat. PloS One.

[15] Jones BC, DeBruine LM, Main JC, Little AC, Welling LL, Feinberg DR, Tiddeman BP (2010). Facial cues of dominance modulate the short-term gaze-cuing effect in human observers. Proc. R. Soc. B Biol. Sci..

[16] Ohlsen G, Van Zoest W, Van Vugt M (2013). Gender and facial dominance in gaze cuing: Emotional context matters in the eyes that we follow. PloS One.

[17] Olivola CY, Todorov A (2010). Elected in 100 milliseconds: Appearance-based trait inferences and voting. J. Nonverbal Behav..

[18] Wilson JP, Rule NO (2015). Facial trustworthiness predicts extreme criminal-sentencing outcomes. Psychol. Sci..

[19] Strayer FF, Trudel M (1984). Developmental changes in the nature and function of social dominance among young children. Ethol. Sociobiol..

[20] Cogsdill EJ, Todorov AT, Spelke ES, Banaji MR (2014). Inferring character from faces: A developmental study. Psychol. Sci..

[21] Terrizzi BF, Brey E, Shutts K, Beier JS (2019). Children’s developing judgments about the physical manifestations of power. Develop. Psychol..

[22] Charlesworth TE, Hudson SKT, Cogsdill EJ, Spelke ES, Banaji MR (2019). Children use targets’ facial appearance to guide and predict social behavior. Develop. Psychol..

[23] Jessen S, Grossmann T (2016). Neural and behavioral evidence for infants' sensitivity to the trustworthiness of faces. J. Cognit. Neurosci..

[24] Quinn PC, Uttley L, Lee K, Gibson A, Smith M, Slater AM, Pascalis O (2008). Infant preference for female faces occurs for same-but not other-race faces. J. Neuropsychol..

[25] Quinn PC, Yahr J, Kuhn A, Slater AM, Pascalis O (2002). Representation of the gender of human faces by infants: A preference for female. Perception.

[26] Sakuta Y, Kanazawa S, Yamaguchi MK (2018). Infants prefer a trustworthy person: An early sign of social cognition in infants. PloS One.

[27] Thomsen L, Frankenhuis WE, Ingold-Smith M, Carey S (2011). Big and mighty: Preverbal infants mentally represent social dominance. Science.

[28] Pun A, Birch SA, Baron AS (2016). Infants use relative numerical group size to infer social dominance. Proc. Natl. Acad. Sci..

[29] Meng X, Nakawake Y, Nitta H, Hashiya K, Moriguchi Y (2019). Space and rank: Infants expect agents in higher position to be socially dominant. Proc. R. Soc. B.

[30] Mascaro O, Csibra G (2012). Representation of stable social dominance relations by human infants. Proc. Natl. Acad. Sci..

[31] Mascaro O, Csibra G (2014). Human infants’ learning of social structures: The case of dominance hierarchy. Psychol. Sci..

[32] Bas J, Sebastian-Galles N (2021). Infants' representation of social hierarchies in absence of physical dominance. Plos One.

[33] Thomas AJ, Thomsen L, Lukowski AF, Abramyan M, Sarnecka BW (2018). Toddlers prefer those who win but not when they win by force. Nat. Hum. Behav..

[34] Thomas AJ, Sarnecka BW (2019). Infants choose those who defer in conflicts. Curr. Biol..

[35] Hawley PH (1999). The ontogenesis of social dominance: A strategy-based evolutionary perspective. Develop. Rev..

[36] Fiske ST, Cuddy AJC, Glick P (2007). Universal dimensions of social cognition: Warmth and competence. Trends Cognit. Sci..

[37] Hall JA, Coats EJ, LeBeau LS (2005). Nonverbal behavior and the vertical dimension of social relations: A meta-analysis. Psychol. Bull..

[38] Dovidio JF, Ellyson SL, Keating CF, Heltman K, Brown CE (1988). The relationship of social power to visual displays of dominance between men and women. J. Personality Social Psychol..

[39] Sutherland CA, Young AW, Mootz CA, Oldmeadow JA (2015). Face gender and stereotypicality influence facial trait evaluation: Counter-stereotypical female faces are negatively evaluated. Br. J. Psychol..

[40] Montepare JM, Dobish H (2003). The contribution of emotion perceptions and their overgeneralizations to trait impressions. J. Nonverbal Behav..

[41] Ramsey JL, Langlois JH, Marti NC (2005). Infant categorization of faces: Ladies first. Develop. Rev..

[42] Grossmann T, Striano T, Friederici AD (2007). Developmental changes in infants’ processing of happy and angry facial expressions: A neurobehavioral study. Brain Cognit..

[43] Lundqvist D, Flykt A, Öhman A (1998). Karolinska directed emotional faces. PsycTESTS Dataset.

[44] Todorov, A., & Oh, D. The structure and perceptual basis of social judgments from faces. in *Advances in Experimental Social Psychology* (Vol. 63, pp. 189–245). (Academic Press, 2021).

[45] Todorov A, Oosterhof NN (2011). Modeling social perception of faces [social sciences]. IEEE Signal Process. Mag..

[46] Scherf KS, Scott LS (2012). Connecting developmental trajectories: Biases in face processing from infancy to adulthood. Develop. Psychobiol..

[47] Barrera ME, Maurer D (1981). Discrimination of strangers by the three-month-old. Child Develop..

[48] Fagan JF (1976). Infants' recognition of invariant features of faces. Child Develop..

[49] Sugden NA, Moulson MC (2019). These are the people in your neighbourhood: Consistency and persistence in infants’ exposure to caregivers’, relatives’, and strangers’ faces across contexts. Vision Res..

[50] Hess U, Adams R, Kleck R (2005). W20o may frown and who should smile? Dominance, affiliation, and the display of happiness and anger. Cognit. Emotion.

[51] Lin C, Keleş Ü, Adolphs R (2021). Four dimensions characterize comprehensive trait judgments of faces. Nat. Commun..

[52] Zebrowitz LA, Wang R, Bronstad PM, Eisenberg D, Undurraga E, Reyes-García V, Godoy R (2012). First impressions from faces among US and culturally isolated Tsimane’ people in the Bolivian rainforest. J. Cross-Cultural Psychol..

[53] Sutherland CAM, Liu X, Zhang L, Chu Y, Oldmeadow JA, Young AW (2018). Facial first impressions across culture: Data-driven modeling of Chinese and British perceivers' unconstrained facial impressions. Personality Social Psychol. Bull..

[54] Ybarra O, Chan E, Park H, Burnstein E, Monin B, Stanik C (2008). Life's recurring challenges and the fundamental dimensions: An integration and its implications for cultural differences and similarities. Eur. J. Social Psychol..

[55] LaBarbera JD, Izard CE, Vietze P, Parisi SA (1976). Four-and six-month-old infants' visual responses to joy, anger, and neutral expressions. Child Develop..

[56] Vaish A, Grossmann T, Woodward A (2008). Not all emotions are created equal: The negativity bias in social-emotional development. Psychol. Bull..

[57] Deen B, Richardson H, Dilks DD, Takahashi A, Keil B, Wald LL (2017). Organization of high-level visual cortex in human infants. Nat. Commun..

[58] Livingstone MS, Vincent JL, Arcaro MJ, Srihasam K, Schade PF, Savage T (2017). Development of the macaque face-patch system. Nat. Commun..

[59] Miao C, Li X, Derrington E, Moisan F, Li Y, Dreher JC (2022). ERPs responses to dominance features from human faces. Sci. Rep..

[60] Breton A, Jerbi K, Henaff M-A, Cheylus A, Baudouin J-Y, Schmitz C, Krolak-Salmon P, Van der Henst J-B (2014). Face the hierarchy: ERP and oscillatory brain responses in social rank processing. PLoS ONE.

[61] Breton A, Ligneul R, Jerbi K, George N, Baudouin J-Y, Van der Henst J-B (2019). How occupational status influences the processing of faces: An EEG study. Neuropsychologia.

[62] De Haan MD, Pascalis O, Johnson MH (2002). Specialization of neural mechanisms underlying face recognition in human infants. J. Cognit. Neurosci..

[63] Halit H, De Haan M, Johnson MH (2003). Cortical specialisation for face processing: Face-sensitive event-related potential components in 3-and 12-month-old infants. Neuroimage.

[64] Smith ER, DeCoster J (2000). Dual-process models in social and cognitive psychology: Conceptual integration and links to underlying memory systems. Personality Social Psychol. Rev..

