# Supplementary material for: Toddlers’ sensitivity to dominance traits from faces
Source: Sci Rep. 2023 Dec 15;13:22292. doi: 10.1038/s41598-023-49385-7 (PMC10721615; doi:10.1038/s41598-023-49385-7)
Supplement: Supplementary file 1 — Supplementary Information. [file 41598_2023_49385_MOESM1_ESM.docx]

**Toddlers’ sensitivity to dominance traits from faces**

Cristina-Ioana Galusca (1)(2), Martial Mermillod (2), Jean-Claude Dreher (3), Jean-Baptiste van der Henst (4), and Olivier Pascalis (1)(2)

**Affiliations:**

(1) Laboratoire de Psychologie et NeuroCognition,Université Grenoble Alpes, France

(2) Centre National de la Recherche Scientifique, Grenoble, France

(3) CNRS-Institut de Sciences Cognitives Marc Jeannerod, UMR5229, Neuroeconomics, Reward, and Decision Making Laboratory, Lyon, France

(4) Université Claude Bernard Lyon 1, CNRS, INSERM, CRNL U1028 UMR5292 Trajectoires

**Supplementary Materials**

**S1**. Here we summarise the results of the Bayesian analyses for the spontaneous visual preference difference scores for Experiments 1-3. For this analysis we used the Bayesian t-test framework by Jeffreys (1961, see also Rouder et al. 2009), to test the hypothesis that toddlers displayed longer looking times at the dominant faces. The data was analysed with JASP (JASP Team, 2019). The null hypothesis postulates that there is no difference in looking time between the dominant and non-dominant face and therefore H0 ∶ δ = 0. The two-sided alternative hypothesis states that difference scores are different from 0, and participants’ looking times are different for the dominant and non-dominant face. As in the main manuscript, we analysed the data for each face type, age and orientation separately.

**Experiment 1 - Artificial Faces**

**18 months**. For upright faces, the Bayesian t-test against the test value = 0 shows a Bayes factor of 1.83, considered moderate evidence in favour of the hypothesis that toddlers did not look at the dominant and non-dominant artificial faces for an equal amount of time (i.e., difference scores differed from 0). The error percentage is < 0.001%, which indicates great stability of the numerical algorithm used to obtain this result.

For inverted faces, the Bayes factor is 6.59, considered moderate to strong evidence in favour of the single-point hypothesis that toddlers attended equally long to the dominant and non-dominant inverted artificial faces. The error percentage is < 0.001%, indicating great stability of the numerical algorithm used to obtain this result.

**24 months**. For upright faces, the Bayesian t-test against the test value = 0 shows a Bayes factor of 2.042, considered weak evidence in favour of the hypothesis that toddlers did not look at the dominant and non-dominant artificial faces for an equal amount of time (i.e., difference scores differed from 0). The error percentage is < 0.001%, which indicates great stability of the numerical algorithm used to obtain this result.

For inverted faces, the Bayes factor is 1.344, considered weak evidence in favour of the single-point hypothesis that toddlers attended equally long to the dominant and non-dominant inverted artificial faces. The error percentage is < 0.001%, indicating great stability of the numerical algorithm used to obtain this result.

**Experiment 2 - Male Faces**

**18 months**. For upright faces, the Bayesian t-test against the test value = 0 shows a Bayes factor of 6.916, considered moderate evidence in favour of the single-point hypothesis that 18-month-old toddlers attended equally long to the dominant and non-dominant upright male faces. The error percentage is < 0.001%, which indicates great stability of the numerical algorithm used to obtain this result.

For inverted faces, the Bayes factor is 6.953, considered moderate evidence in favour of the single-point hypothesis that toddlers attended equally long to the dominant and non-dominant inverted male faces. The error percentage is < 0.001%, indicating great stability of the numerical algorithm used to obtain this result.

**24 months**. For upright faces, the Bayesian t-test against the test value = 0 shows a Bayes factor of 2.701, considered weak evidence in favour of the single-point hypothesis that toddlers attended equally long to the dominant and non-dominant upright male faces. The error percentage is < 0.001%, which indicates great stability of the numerical algorithm used to obtain this result.

For inverted faces, the Bayes factor is 7.699, considered moderate evidence in favour of the single-point hypothesis that toddlers attended equally long to the dominant and non-dominant inverted male faces. The error percentage is < 0.001%, indicating great stability of the numerical algorithm used to obtain this result.

**Experiment 3 - Female Faces**

**18 months**. For upright faces, the Bayesian t-test against the test value = 0 shows a Bayes factor of 7.245, considered moderate evidence in favour of the single-point hypothesis that toddlers attended equally long to the dominant and non-dominant upright female faces. The error percentage is < 0.001%, which indicates great stability of the numerical algorithm used to obtain this result.

For inverted faces, the Bayes factor is 7.256, considered moderate evidence in favour of the single-point hypothesis that toddlers attended equally long to the dominant and non-dominant inverted female faces. The error percentage is < 0.001%, indicating great stability of the numerical algorithm used to obtain this result.

**24 months**. For upright faces, the Bayesian t-test against the test value = 0 shows a Bayes factor of 1.132, considered weak evidence in favour of the hypothesis that toddlers did not look at the dominant and non-dominant female faces for an equal amount of time (i.e., difference scores differed from 0). The error percentage is < 0.001%, which indicates great stability of the numerical algorithm used to obtain this result.

For inverted faces, the Bayes factor is 6.491, considered moderate evidence in favour of the single-point hypothesis that toddlers attended equally long to the dominant and non-dominant inverted female faces. The error percentage is < 0.001%, indicating great stability of the numerical algorithm used to obtain this result.
